# Conceptualizing Youth Participation in Children’s Health Research: Insights from a Youth-Driven Process for Developing a Youth Advisory Council

**DOI:** 10.3390/children6010003

**Published:** 2018-12-28

**Authors:** Krishna Arunkumar, Drew D. Bowman, Stephanie E. Coen, Mohammad A. El-Bagdady, Christina R. Ergler, Jason A. Gilliland, Ahad Mahmood, Suraj Paul

**Affiliations:** 1 Human Environments Analysis Laboratory, Western University, London, ON N6A 5C2, Canada; karunkum@uwo.ca (K.A.); dbowman7@uwo.ca (D.D.B.); scoen@uwo.ca (S.E.C.); melbagda@uwo.ca (M.A.E.-B.); christina.ergler@otago.ac.nz (C.R.E.); amahmo46@uwo.ca (A.M.); spaul59@uwo.ca (S.P.); 2 Department of Geography, Western University, London, ON N6A 5C2, Canada; 3 Children’s Health Research Institute, London, ON N6C 2V5, Canada; 4 Department of Geography, University of Otago, Dunedin 9054, New Zealand; 5 Department of Paediatrics, Western University; London, ON N6A 5C1, Canada; 6 Faculty of Health Sciences, Western University; London, ON N6A 3K7, Canada; 7 Department of Epidemiology & Biostatistics, Western University; London, ON N6A 5C1, Canada

**Keywords:** children’s health, health geography, healthy cities, healthy communities, participation, participatory health research, place-based health research, youth advisory council, youth

## Abstract

Given the power asymmetries between adults and young people, youth involvement in research is often at risk of tokenism. While many disciplines have seen a shift from conducting research on youth to conducting research with and for youth, engaging children and teens in research remains fraught with conceptual, methodological, and practical challenges. Arnstein’s foundational Ladder of Participation has been adapted in novel ways in youth research, but in this paper, we present a new rendering: a ‘rope ladder.’ This concept came out of our youth-driven planning process to develop a Youth Advisory Council for the Human Environments Analysis Laboratory, an interdisciplinary research laboratory focused on developing healthy communities for young people. As opposed to a traditional ladder, composed of rigid material and maintaining a static position, the key innovation of our concept is that it integrates a greater degree of flexibility and mobility by allowing dynamic movement beyond a 2D vertical plane. At the same time, the pliable nature of the rope makes it both responsive and susceptible to exogenous forces. We argue that involving youth in the design of their own participatory framework reveals dimensions of participation that are important to youth, which may not be captured by the existing participatory models.

## 1. Introduction

Given the inherent power asymmetries between adults and young people, youth involvement in academic research is often at risk of tokenism, as per Arnstein’s Ladder of Participation [1,2,3]. While many disciplines have seen an epistemological shift from conducting research on youth to conducting research with and for youth, engaging children and teens in research remains fraught with conceptual, methodological, and practical challenges [4,5,6,7,8,9,10,11]. In this paper, we present a new rendering of Arnstein’s Ladder, which originally was established to encourage an enlightened dialogue on citizenship participation and to discuss the extent of citizens’ decision making power, derived from our critical reflections on our youth-driven development process for creating a Youth Advisory Council (YAC) for the Human Environments Analysis Laboratory (HEAL). The HEAL is an interdisciplinary research laboratory, which embraces cross-sector collaborations for developing healthy communities for children and youth, based in the Department of Geography at Western University in London, Ontario, Canada. 

Although scholars are increasingly aware of the benefits that youth bring to the table for informing and improving outcomes of research [8,12,13], there are often power imbalances and tokenistic practices that result from this inclusion of young people [1,2]—what Arnstein has called an illusion of participation [3]. When it comes to youth, genuine participation is often conceptualized as youth-initiated [1], but what this means is less clear. YACs are one type of participatory mechanism to involve youth in academic research; however, there are currently few frameworks to guide researchers in the development and maintenance of a successful YAC [14]. Our work addresses this void by detailing our youth-driven YAC development process and the new youth-informed iteration of Arnstein’s Ladder that we derived from it. As opposed to Arnstein’s traditional ladder, composed of rigid material and maintaining a static position, the key innovation of our ‘rope ladder’ concept is that it integrates a greater degree of flexibility and mobility than past approaches by allowing dynamic movement beyond a 2D vertical plane; the rope can be swung (up, down, laterally, diagonally), knotted, and looped. This framework contributes to the theoretical foundation of participatory approaches by building on and adapting key insights from Arnstein’s original ladder to a contemporary research context with youth as architects of participatory constructs. Therefore, the aims of this paper are threefold. First, we discuss our experiences of establishing a YAC in the HEAL to offer much-needed guidance on the development and use of YACs as a participatory approach with young people. Second, we present a new model of participation—the rope ladder—as an outcome of our youth-driven YAC development process, to help guide others that are seeking to cultivate youth–adult collaborations. Third, we suggest that the rope ladder can also be used as a potential tool for evaluating and thinking through the pitfalls and benefits in participatory collaborations with youth.

To situate our rope ladder model, we begin by laying out why youth participation matters in research and practice. We then review and critique some of the existing models of youth participation, and go on to show how YACs can be one important modality to realize youth participation in research. Next, we provide background about our research on children’s health and wellbeing at the HEAL, before we detail our youth-driven YAC development process, highlighting the iterative dynamics of this engagement with youth. Finally, we present our rope ladder model, showing how building flexibility into participatory structures can allow for multidimensional modes of engagement that benefit everyone that is involved.

## 2. Background: Conceptualizing Meaningful Youth Participation

### 2.1. Integrating Youth Perspectives in Research and Practice 

The United Nations Convention on the Rights of the Child requires researchers and decision-makers to listen and give due weight to the voices of children on matters that affect them [15]. Nevertheless, meaningful working relationships between professionals (e.g., health promoters, planners, city decision makers) and children have been limited [12,16,17,18,19,20,21]. This is despite the acknowledgement by governments and policy makers of the need to address children’s political, legal and social rights through policy and in practice. Young adults make up a large proportion of our communities, and are greatly susceptible to change within their environments, but their views are often not considered in the research and planning processes [19,21,22]. Frank argues that as key stakeholders within society, young adults should be given the opportunity to have an active voice in community-related research, as these decisions often directly affect the spaces in which they live, and in turn, their health [7]. However, practitioners often find it difficult to work with young people of different ages and abilities (even when they are willing), not knowing how to interpret and respond to children’s experiences and suggestions in meaningful and effective ways [13,16,23]. Researchers report on issues around logistics, time commitment or data quality when involving young people as researchers [24,25,26]. As a consequence, many researchers and decision-makers turn away from involving young people in decision-making processes. This lack of engagement often leads to young people expressing feelings of inferiority and lack of decision-making power within their communities compared to adults [22]. However, these adult-led, decision-making processes directly affect the areas of concern that are addressed in research, and consequently, the spaces in which young people live, work, play, and enjoy themselves, with consequences for their quality of life, health, and wellbeing [7,22]. 

Young adults are immersed within their local neighborhoods and communities on a daily basis, and thus they deserve to be engaged in the planning processes, considering that they may be particularly vulnerable to place-specific effects on health [27]. This idea is supported by evidence brought forth by Osborne and colleagues, who highlight the vulnerabilities of youth, considering their local built environments influence their health-related behaviours and quality of life [22]. Horgan and colleagues also argue that the geographic and environmental contexts that youth are exposed to can be considered “thinners” or “thickeners” of youth influence [28] (p. 279). For instance, some aspects of the built environment can enhance the agency of youth, thus expanding or thickening their power, whereas other features of the community act as a restricting force, thus thinning their influential capacity. Engaging youth in planning and community-related decisions, and allowing them to partake in the process of placemaking, can foster youth attachment to their local environments, and to the places within these geographic areas [27].

Incorporating the voices and unique perspectives of young people also allows for the generation of research findings and outcomes that align with the views of the youth themselves [21]. The benefits associated with engaging youth in research processes include increased feelings of belonging and ownership within their communities, the development of more “youth-friendly spaces”, enhanced social development, and social and intergenerational connectedness [22] (p. 357). Young people can also gain an awareness of their own democratic rights as citizens, with positive impacts on the attachment to their local environments [29,30]. Moreover, youth–adult partnerships can provide opportunities for ‘mutual learning’ between young people and members of diverse communities to co-create healthier spaces and more liveable urban environments for people of all ages [2,14,31]. On a broader scale, the inclusion of young adults in research can allow for more efficient and effective community-level outcomes, including appropriate policies, practices, and research programs that directly improve the lives of this sometimes marginalized demographic [32].

Although such reciprocal adult–youth partnerships in research are often idealized [8,9], it is often not achieved in practice; adults attempt to integrate youth into the research process without critically considering how this youth–adult dynamic will function, resulting in limited decision-making and participation on the part of children and young adults [1,21]. However, by denying young people opportunities to participate properly in decision-making processes related to their quality of life and the social and physical wellbeing of their communities, important prospects are missed for mobilizing inclusive and collective action on social, community, and environmental change [29]. Thus, adopting a youth participatory approach will enhance knowledge translation and narrow the knowledge disconnect between adults and youth, who both have distinct and diverse views, for improved planning and health outcomes [33]. Nonetheless, good practice guidelines for adult–youth partnerships in research projects are scarce. Therefore, we provide a potential guiding framework for involving young people in local research and planning decision-making processes that can enhance their and their communities’ health and wellbeing. We propose to use the metaphor of a rope ladder for establishing meaningful and effective collaborations between young people and adults. Such a ladder, as we outline below, presents a way to address the needs of adults and young people, as well as shortfalls in Arnstein’s ladder.

### 2.2. Continuously Climbing or Falling Short? Ladder(s) of Youth Participation

Sherry Arnstein, a US based city planner, developed the typology of a ladder of participation in 1969 in response to the popularity of citizen participation in decision-making processes. She noticed that the term participation was used in diverse ways, and for many purposes, which led her to critique some of the existing practices of participation. Her typology in form of an ascending ladder with nonparticipation at the bottom and genuine citizen power as the highest rung reflects the varying degrees of participation. Her intent was to unravel the processes that aimed at ‘educating’ or ‘curing’ participants, with the end goal of buying into and agreeing to the ideas of people in power. The model was seen as a critique of ‘tokenistic’ approaches, whereas genuine citizen power was considered as good practice [3].

Since the advent of Arnstein’s foundational Ladder of Participation, the concept has informed and been adapted in various ways to conceptualize young people’s participation [3]. Hart made an early intervention by adapting Arnstein’s ladder to reflect varying degrees of youth (non)participation, notably re-framing Arnstein’s concepts to represent the relative balance of power among youth and adults [1]. Similarly, Francis and Lorenzo advocate for a “proactive realm” to youth participation within the field of environmental planning and design, which operates from the perspective of “planning with children” rather than “planning by children” [34] (p. 157). Checkoway conceptualizes youth participation simply as when youth are actively engaged and have real influence [19]. Participation from this perspective can be assessed according to specific objectives, outcomes, and evaluative criteria. This includes not only how participation affects youth, but how the involvement of youth impacts society—yet, Checkoway notes that few studies have evaluated the effects of participation empirically [19]. Instead, discussions have centered on the refinement of the ladder concept or the proposal of alternative models.

These alternative concepts are often built on the premise that there should be various types of participation [10,35]. For instance, Wong et al. introduce “five types of participation” that describe unique levels of youth engagement and involvement within the research process [6] (p. 104). Similarly, Checkoway and Richards-Schuster propose a model that is specific to the research roles of young people as ranging from youth as subjects, to consultants, to partners, to directors across the various phases of research, showing varying degrees of the balance of intergenerational involvement across roles [29]. Hart [35] notes that circle and fountain-like models of participation have been explored by others [36,37]. Ergler, staying closer to the original ladder concept, proposed a ‘jungle gym’ model that allowed for more varied levels and forms of participation, disrupting the hierarchy of a typical ladder [10]. Despite these innovations, most ladder concepts remain sequential and hierarchical [35,38] and with attention focused on the meaning and positioning of the rungs. Even Arnstein accepted that the realities of participation are more nuanced than the domains she presents, noting that, “there might be 150 rungs with less sharp and ‘pure’ distinctions among them” [3] (p. 217).

As opposed to Arnstein’s ladder, with citizen control at the top, Hart later clarified that his youth-focused ladder recognizes the limits on youth autonomy by noting that the highest rung is not youth control [35]. Hart comments that while his adaption of the ladder was not meant to imply that forms of participation occur in sequence, there will always be some degree of hierarchy in participation, as not all forms of participation are equal [35]. At the same time, the intention of Hart’s (1997) ladder is to afford young people opportunities to participate at the highest rung of their choosing. In contrast, Jensen [39], as discussed in Hart [35], has even suggested that the rungs of the ladder be described as different forms rather than different levels of participation, which can also be read into Hart’s proposal to view his ladder in a ‘scaffold metaphor:’ “whereas the ladder metaphor is usually used to characterize only child–adult relations, the scaffold metaphor can be thought of as a mutually reinforcing structure where all people, including adults and children of different abilities, help each other in their different climbing goals” [35] (p. 21). This is also consistent with Ergler’s argument for more mutually reinforcing infrastructure among youth and adults, which supports this notion of a dynamic, less one-directional ladder, and thus, argues against always holding the top rung of Arnstein’s ladder as the ‘gold standard’ of participation [9,10]. Past research has revealed that youth may prefer to have flexibility in the terms of their participation, and be able to move fluidly between the rungs of participation, depending on various factors, such as their interests or experiences [10]. Ergler’s ‘jungle-gym’ model of participation works to address this issue by providing multi-directionality within its structure and allowing youth to self-determine their level of involvement. Nonetheless, we note that dominant traditional depictions of the ladder connote a one-way directionality of participation; that is, up is best. Moreover, Malone and Hartung critique existing concepts of youth participation, as they are ultimately adult-created, which raises a fundamental question about how we conceptualize participation in the first place, if youth are not part of concept development [38].

These critiques highlight the need for youth involvement in the development of our models of participation, as well as concepts that allow for flexibility, adaptability, and which consider the context of youth’s everyday lives. Thus, we advocate for a more dynamic, less static rope ladder model and we base our model on the following premises:

1. Moving away from adult-created research processes to ensure genuine participation, with youth being part of developing a meaningful and genuine model of participation [38];
2. Ensuring diversity of participants (e.g., age, grade, background, school, socio-economic status etc.) for creating a relevant model of participation [40];
3. Creating a flexible and adaptable approach to participation that accounts for the context of youth’s everyday life, and allows the self-determination of youth for their level of involvement in decision-making and participation processes at any stage of the research/participation process (moving fluidly between rungs) [9];
4. Recognizing the cultural context in which models of participation are set, and how the external contextual influences such as local political climate or the availability of resources can influence the dynamics of participation [35,41];
5. Creating mechanisms for how adults relinquish power and are accountable to youth (e.g., ensuring the transparency of decision-making processes) in a way that speaks to young people’s needs and visions [1,29];
6. Creating trustful youth–adult partnerships; and
7. Ensuring that the model can address the complex relationships, needs, and visions of youth and adult partners, and can account for the multifaceted, flexible nature of youth advisory councils and adult–youth partnerships.

### 2.3. Youth Advisory Councils as Mechanisms of Participation

Youth Advisory Councils (YACs) are a promising participatory mechanism to integrate youth perspectives and experiences in research and practice. Bogar and colleagues, for example, observed that through adult–youth collaboration in the form of a YAC, young adults are able to generate insightful and unique perceptions about the complex nature of their local environment [27]; such generation of ‘new knowledge’ reiterates the importance of achieving demographic diversity, specifically youth involvement and empowerment, in research planning and implementation [42,43]. YACs thus provide one way to potentially foster more inclusive approaches for engaging youth throughout the entire research process, or at least for gaining their advice on research projects—an area that remains a challenge for much participatory research [9,44].

At the same time, as with other forms of community-based youth research, YACs could still end up being adult-controlled without accountability, and so there is still work to be done to mitigate these power imbalances in the context of youth research [3,10]. There is also the danger that YACs can be set up by adults in ways that end up ticking a box for participatory performance indicators, without any substantive role for youth to affect outcomes [30]. Others caution that youth participation often involves short-term projects, and that the sometimes temporary nature of youth advisory councils could pose a barrier to achieving effective community research outcomes that are representative of youth’s perspectives [45]. To counter these pitfalls, Richards-Schuster, reporting on the work of a teen advisory council focused on youth grant-making, puts forth four core principles of success, including valuing teens as advisors in the research process, providing them with opportunities to engage in research, ongoing training, and education to support their involvement, and the requirement that adult leaders trust the views of young people [46]. The authors suggest ensuring that YACs focus on leveraging strengths, not only ‘fixing’ youth problems, as well as ensuring that YACs afford opportunities for youth growth [46,47].

Keeping these potential pitfalls in mind, and the potential for YACs to meaningfully involve youth and to ensure beneficial outcomes for all parties, we focused on a YAC as the modality for integrating youth perspectives in our research. We hope that insights from our method of youth engagement will be used as a guiding framework by other researchers, to assist them in developing their own unique version of a youth advisory council that fosters genuine and active youth participation, not simply the illusion of it.

## 3. Creating Our Youth Advisory Council: A Youth-Driven Development Process

Our interdisciplinary research at the HEAL collectively aims to improve the health of young people. We study how a diversity of environmental conditions shapes youth health issues, ranging from mental health and quality of life [48,49], to participation in physical activity and active transportation [50,51], to nutrition and food purchasing patterns [52,53]. The impetus for our YAC is rooted in our values as a lab. We believe that youth perspectives are necessary to ensure the integrity and quality of our research, so that our research has the greatest potential to improve the health and wellbeing of youth. In this way, the YAC advances our collective vision of affecting positive change through our research and improving the health of young people within the community.

The HEAL’s YAC—called HEALYAC—was developed with the historical power inequities among youth and adults in mind and strives to diminish the adult–youth hierarchy within the research process, through the creation of a guiding framework for the meaningful inclusion of youth. We recognize the importance of adult–youth collaboration throughout all stages of the research process, to be able to effectively plan, implement, and evaluate research in ways that positively impact community health. The HEAL aims to fosters an opportunity role structure for youth, that supports growth through learning and acquired knowledge; the HEAL has experienced members who can offer guidance and support to young adults throughout their engagement in the HEALYAC and the research process. 

In establishing a YAC for the HEAL, our purpose was to integrate youth voices, perspectives, and expertise into our research on young people’s health and wellbeing. We aimed to overtly position youth input as being integral to shaping both the wider strategic directions and priorities of our lab, and decision-making at the level of specific projects. We also envisioned our relationship with the HEALYAC as a two-way street: the youth advise HEAL projects and directions, ensuring relevance and resonance to strengthen our work, and the youth receive leadership experience, research training, and education [6,54,55]. This bi-directional relationship functions as a key component of our HEALYAC, where the HEAL has a priority of ‘giving back’ to the youth (through workshops and educational opportunities) in appreciation of their assistance and valuable input. The development of our HEALYAC supports a proactive realm of participation [34] by bringing youth to the table before a research project is even implemented. Therefore, our HEALYAC will add to what Horgan et al. [28] refers to as the ‘thickening’ of youth agency within the London community, and enable them to actively participate in decisions that directly affect themselves and their neighborhoods. Rather than youth being caught ‘in the middle’ of research, between adults and community-related decisions that impact their quality of life, we are pushing for a shift in research; we envision the research process to be more of a partnership with youth, with the ideology of ‘sharing of power’ instead of imposing power upon them [42,56,57].

In developing our YAC, our process was characterized by what Derr has called for as a “deepening of the process, from one that views participation as a hierarchical power relation to one that moves the process into a dialogue with mutual benefits and exchange” [4] (p. 120). As opposed to creating a staff- or adult-driven model for our YAC, we made a conscious decision in our YAC development process to ensure that the youth had lead roles in the development of the council. In doing so, we respond to Malone and Hartung’s call that theories of youth participation “should be generated from the field rather than applied from adult-centered theory building” [38] (p. 26). This partnership, as an alliance between adults and youth, helped us to move toward developing a reciprocal relationship from the beginning, as opposed to a more authoritative one [1]. Such an adult–youth co-learning partnership allowed for the adults to guide the youth, acting as “natural mentors” for the young people, but keeping them at the heart of the decision-making process [58] (p. 173). In other words, we conceptualized a youth-driven process as being directed by youth, with the support of HEAL staff and students, so that the development process itself provided opportunities for mentorship and youth leadership and research skills development [58]. A youth member of our YAC Development Team reflects on this experience in Box 1. On a more theoretical level, this process led to the development of the model of a rope ladder. We realized that a new model of participation is warranted to reflect the complexity of our experiences and needs during the YAC development process.

Box 1 **Reflections on the YAC Development Process.**

Being a high school student among researchers and other adults was an intimidating experience at the beginning of this project. It’s an interesting power dynamic and it’s not often that adults rely on youth for their opinions. I was afraid of making a mistake or providing the “wrong answer” so I chose to be careful about what I shared. This not only curbed the development of many ideas I had, but also limited my participation in discussions. Over time, I acclimatized to the lab and its members, which made it easier to communicate and become an active participant. This feeling of being inexperienced was a great personal barrier I was required to overcome during consultations and even interviews with future YAC members. Having adults who not only trusted me but also supported my opinions, greatly aided me in overcoming this obstacle and allowed me to gain confidence along the way. I not only worked through personal barriers but also gained new skills through the development of this YAC. Reading and understanding academic papers is not only a foreign concept in high school, but it is a skill that requires years of practice. Receiving exposure to academic literature not only proved to be a prominent starting point for our development of the YAC but also introduced a very important side of academia that we will encounter in our post-secondary endeavors. However, literature was only the beginning of everything I learned during my time in the lab. I gained knowledge about research as a whole in terms of ethical considerations, grants and writing research papers. Creating the council went from something that would ideally coincide with our co-op terms and end in June, to a project that is still ongoing. Not only were we introduced to new concepts to consider in order for our YAC to function successfully, we were also editing and adding to our terms of reference. It was a constant loop of creating, consulting and fixing that is still progressing. Altogether, creating this YAC not only helped me overcome personal obstacles but also proved to be an incredibly unique experience that introduced me to the world of academia. It made me realize how essential youth voice is to research and how much they could gain from an experience similar to mine. This is why we created the YAC, to elevate underrepresented voices in research and to allow youth to have a unique opportunity to express their opinions.

~ Krishna Arunkumar, Grade 12

**Figure 1:**
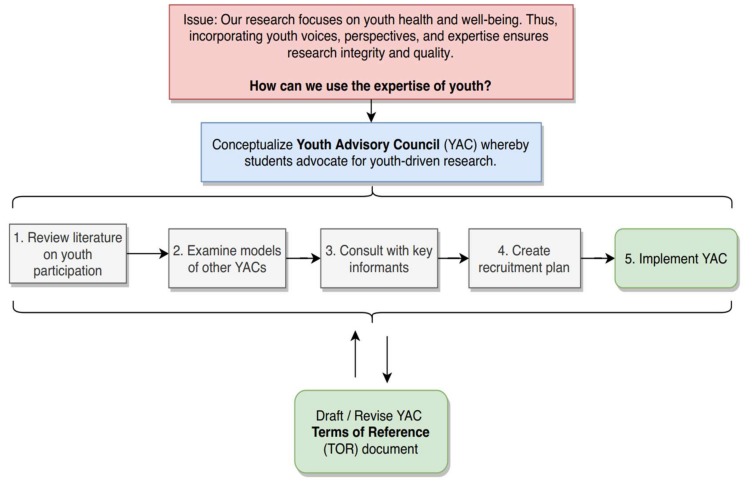
Schematic of our process for developing a Youth Advisory Council (YAC).

In January 2018, we invited three high school students (K.A., A.M., S.P.) who were carrying out practicum placements at the HEAL, as part of a high school for-credit course, to take on the role of the leaders of our YAC Development Team. Students were encouraged to exercise their original ideas and creativity for the YAC, with the ultimate goals of (1) creating a HEALYAC Terms of Reference that would provide an initial framework for the YAC, and (2) recruiting and launching the inaugural YAC. Figure 1 illustrates the various phases and iterative nature our YAC development process, from initial research to launch. To achieve these goals, the YAC Development Team engaged in an iterative two-part process, where staff roles in this process were conceived as being facilitative. For example, the staff pointed students to resources, helped to facilitate academic protocols (such as ethics approval), and provided guidance and feedback as needed.

First, the students worked to base their developmental decisions in academic literature and examples from other YACs. Our youth members, along with staff, critically reviewed academic literature about youth advisory councils and youth participation, as well as terms of reference from other YACs, and applied their findings to drafting our terms of reference. A key part of this task was to critically consider how Arnstein’s ladder could inform our YAC. We engaged in detailed conversations about where on the ladder our YAC would fit. Although the main goal of this adult–youth partnership is to avoid tokenism, the youth decided that there is no ‘ideal rung’ that they wanted to achieve during their involvement in the research process. Rather, there was a consensus that a more flexible approach should be taken, where youth can choose to move up and down the ladder freely, depending on various factors.

Second, based on questions raised in the literature and other YAC models, our team carried out key informant interviews with experts and stakeholders (*n* = 7) with experience in youth advisory councils and participatory approaches to working with youth to gain insight into YAC best practices. These interviews were conceived as a way to integrate first-hand experiences into our own understanding of YAC best practices, and also a way to get expert feedback on our draft terms of reference. One of the youth members reflects on this aspect of our process in Box 2. Our youth team led the development of the interview guide and conducted all interviews, with support from staff co-facilitators. To more systematically assess the recommendations based on our interviews, two students were trained in qualitative coding techniques, and led the initial coding of interview material. The insights gleaned from these interviews were integrated on an ongoing basis in our evolving draft terms of reference. Ethics approval was granted by the Western University’s Non-Medical Research Ethics Board.

Box 2 **Reflections on interviews with key informants.**

We consulted with local experts with experience in youth participation. These consultations involved a discussion about our YAC in hopes to generate new ideas or improve and build upon the basic ideas we have thought about to further develop the YAC. After every consultation, we were left with a plethora of things to add to our draft TOR indicating how important the consultations for developing the YAC. I never realized how many small details we missed. For example, something as small as setting a meeting time seems unconscious, but can be detrimental to the attendance of council members if it is not well thought-out.

~ Suraj Paul, Year 1 Undergraduate

Throughout the process of developing our YAC model, it became clear to us that it was not possible or desirable to pick a rung on Arnstein’s ladder to define the functioning of our YAC. Our youth members voiced a need for much greater flexibility than the ladder allowed. They identified both macro and micro needs for flexibility; that is, for the YAC as a whole to be able to determine the terms of its participation based on the nature of the tasks at hand, as well as for individual members to have agency over the nature of their participation within the group, depending on the work at play. We thus subscribed to “The notion is that there are no single best forms of participation; instead, the rules should be adapted to the group” as well as the individual [19] (p. 343). In relating what we learned from our review of the literature and existing YAC structures, as well as our key informant interviews, back to our HEALYAC terms of reference, we realized that we needed to articulate a new model of participation that was more compatible with our vision for the YAC.

## 4. The Rope Ladder: A New Model for Youth Participation

**Figure 2:**
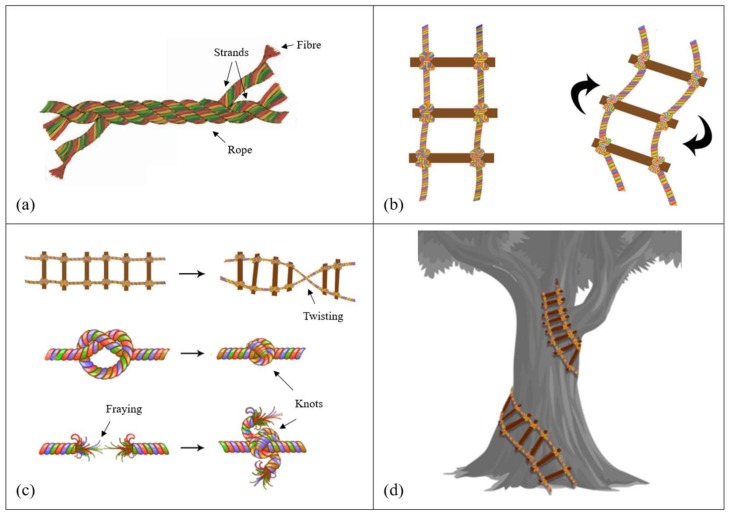
A rope ladder model of youth participation. (**a**) Braiding illustrates how diverse fibers form strands, and strands are braided together to construct the rope; (**b**) Swinging and swaying shows how the rope ladder can move flexibly in multiple directions; (**c**) Knotting and twisting depicts how knots can be employed to fix frays in the rope, and twists can create obstacles; (**d**) Looping shows the circuitous formation the rope ladder can take—rather than straight up and down—which can integrate feedback throughout the process.

As opposed to a traditional ladder, composed of rigid material and maintaining a static position, the key innovation of our concept is that it integrates a greater degree of flexibility and mobility than past approaches, by allowing for dynamic movement beyond a 2D vertical plane; the rope can be braided, swung and swayed (up, down, laterally, diagonally), knotted and twisted, and looped. Figure 2 depicts these functions in each of its four quadrants, respectively. A rope ladder is characterized by multidimensionality; it can move in multiple planes. Here we present the core functional dimensions of our rope ladder model of participation. Rather than focusing on hierarchical rungs that align with different tiers of participation, as per Arnstein’s model, we conceptualize how the various functional capacities of the rope ladder allow for different modes of engagement, driven by youth. By virtue of the flexibility of the rope ladder, youth agency is at the core of our model; youth configure how the rope ladder is deployed in relation to our research lab. Rather than having ‘citizen control’ at the apex, the rope ladder inherently requires action on the part of its users to move the ladder into its desired position or function, be it static or dynamic. This flexibility was important to youth on our development team, because blanket total control was actually antithetical to what they wanted to get out of the YAC—they wanted educational growth and mentorship that allowed them to gain decision-making confidence; blanket ‘citizen control’ from the outset without supportive mentorship was burdensome, rather than productive (see also Box 1). A traditional ladder involves only standing it upright and climbing up or down. At the same time, the pliable nature of the rope can make it both responsive and susceptible to exogenous forces; it may fray. This requires certain preventive or restorative maneuvers on the part of users, such as braiding the rope to further reinforce it, or knotting to repair a break or weak spot.

The strength of rope is typically measured by its flexibility—‘tensile strength’ is a fundamental difference between our ladder and Arnstein’s. Whereas a traditional ladder’s strength depends on the load it can bear before breaking (compressive strength), a rope ladder’s strength is about its capacity to remain intact when stretched (tensile strength). A YAC requires tensile strength if is to continue to function effectively.

### 4.1. Braiding

Braiding is the foundational mechanism by which the rope ladder is held together. Fibers are formed into strands, strands are braided together to make rope, and the rope is what links the rungs. This is where the participatory character of the rope ladder begins, in the base materials. Who selects the fibers? What are they made of? Where do they come from? Diverse fibers make more robust strands and a stronger rope. Braiding distributes the weight-bearing load across multiple strands, so that it does not fall onto only the shortest or weakest. This demonstrates the participatory nature of the council in that each member’s opinions are weighted equally, and responsibility is not piled on to only one or two individuals. There is a tipping point, however—too many strands can make a rope stiff, heavy, and inflexible. Our YAC was conceived as having between 10 and 14 members, to remain strong yet flexible.

When conceptualizing our YAC, we focused on diversity in the composition of our YAC as part of what gives our council its tensile strength (see also Box 4). To do so, we articulated diversity as a core value in our HEALYAC Terms of Reference. We conceived the diversity of the fibers as the identities and experiences of council members, as well as the various roles and skills that members bring to the table. Those that are complementary will help to strengthen the strands by fitting together in a mutually reinforcing way. When we finally invited new YAC members, one aim was to ensure diversity in gender, age, ethnicity, and academic background, to ensure that a diversity of fibers can be braided together over time.

Determining what a participatory platform is ‘made of’ is particularly important when it comes to engaging youth, given that many participatory approaches are still being adult-devised [38]. If we choose the ‘wrong’ materials from the outset, our participatory capacity may be hindered. Inherent in rope as a medium is a ‘coming-togetherness’, which requires a careful consideration of diversity to create an inclusive participatory platform, with optimal tensile strength. Diversity is a crucial consideration in youth participation, because it allows for the inclusion and incorporation of a wide range of unique views and insights from individuals within a community; this component also ensures a more holistic understanding of the desires and needs of young adults within their local environment [46]. Having a diverse group of young adults is also essential, to ensure that social and environmental inequities are not reinforced [59,60]. Yet, previous iterations of Arnstein’s ladder have not contended with diversity as a core issue of participation, instead focusing on the power dynamics between youth and adults without explicitly seeing diversity among youth as a strength to be leveraged in enhancing participation. Horgan et al. [28] also argue that many youth involvement projects are contingent on age and capability, and fail to represent the diversity of youth living within these places. According to Sarkissian and colleagues, diversity is an important feature of any council, but in turn, it is also essential that each member is open-minded and able to listen, learn, and grow from their counterparts [61]. Our experience suggests that diversity be ‘braided into’ participatory platforms from the outset, in thoughtful ways for the benefits of young people and adults. For example, at the HEALYAC orientation, the young council members were asked to share their views on what diversity means to them. They each wrote out their own unique ideas, but came together afterward to discuss their contributions. This activity resulted in increased awareness and appreciation of the diverse perspectives that each member brings to the table. This exercise thus acted as a ‘braiding of views’ and seemed to unify the HEALYAC and emphasize the importance of diversity.

### 4.2. Swinging and Swaying

Sway is movement, but sway is also having power. One of the most distinguishing features of the rope ladder vis-a-vis a traditional ladder is that it can swing and sway in multiple planes. Whereas a traditional ladder can only be placed in one static position, and the user moves either up or down, a rope ladder can be initiated into various movements by the user; the ladder itself can be deployed as a mobility device to swing or sway the user into new positions. This functionality is especially useful when our concept of participation challenges the idealization of the ‘top rung’ of participation. For example, there may be instances when the rope ladder is used to sway out laterally—a direction not afforded by a traditional ladder. A lateral move may enable youth to maintain their level of participation, if desired, while configuring new and different ways to contribute. This function helps to sensitize the ladder to group and individual needs, taking into account the wider context of youths’ everyday lives and commitments; they may not always be able to take on more, but can adjust their participation within the range that suits. With this function, youth ‘have sway’ to negotiate the terms of their engagement. The ladder can also be swung into more or less involved modes of participation. Rather than moving in a straightforward (up and down) fashion, this swinging functionality allows for creativity, flexibility, and improvisation in youth participation. This swinging capacity also allows for the power balance in decision making authority among youth and adults to shift, depending upon the situation. This mobility also makes the rope ladder both responsive and potentially vulnerable to exogenous forces. On the responsive side, given the ease of moving the rope, youth may quickly adapt their participation to changing external circumstances, such as new policies or investments that present new opportunities to advance collective goals. On the negative side, there may be external conditions that test the tensile strength of the rope, or even create friction or frays in ways that may temporarily, or detrimentally, decrease participatory capacity. Nevertheless, there are also possible scenarios where the sheer force of gravity outweighs other forces (in much the same way that adult voices can drown out youth voices), acting to hold the ladder down and ultimately returning it to a rigid vertical plane.

We have built this dynamic movement in our YAC (both as a group and individually) to allow for shifting participation based on comfort, interest level, and internal/external constraints. This changes with respect to both the specific research project and the stage of the research (planning, implementation, evaluation, etc.) When working with youth, it is important to recognize that the YAC may not be their main priority, as they are partaking in various other activities as well. A more flexible approach to youth participation enables the youth to choose when they can achieve the higher-rungs, without qualifying varied participation as being better or worse. Moreover, our youth members challenged the notion that the YAC should always occupy the highest rung of the ladder (what Arnstein called Citizen Control) [9,10]; instead, concluding that the ultimate decision-making authority should rest with the HEAL, but that it requires mechanisms for accountability (see Looping, below).

### 4.3. Knotting and Twisting

Knots can be both assets and liabilities on the rope ladder. On the positive side, knots can be used to extend the ladder by tying on additional rope. Knots can also be used to further reinforce the ladder, or to add new rungs. Knotting can also be seen as a solution; it can function as a way to both fix our mistakes and ‘climb over them’. It is not a coincidence that knots can also be used to climb a rope ladder; overcoming issues and discrepancies can allow us to get a grip on the issue, and once a solution is found, it allows us to step up and over that issue, and onto our next research challenge. If our rope ladder is frayed or cut, this represents a flaw in the structure of the ladder. Knotting is a method that can be used to fix frayed or broken pieces of our rope ladder. Disagreements or discrepancies, like knots, can be solved. Similar to knots as a liability, the ladder can become twisted in ways that inhibit movement along it, or even render it useless. More positively, (un)twisting can be a method to untie knots, or to overcome certain situations. Like the human knot exercise, where individuals must work together to untie themselves, this notion of knotting and twisting is like problem solving and teamwork used to collectively come up with an appropriate solution. When knots and twists constitute barriers or challenges—things that get in the way of the mobility of the ladder—undoing them further strengthens the collective participatory apparatus by freeing up the capacity that was caught up elsewhere. In conceptualizing knots and twists in participation, it is important to think through where productive (helpful) knots are needed, and where potential knots and twists as barriers may occur.

As compared with Arnstein’s ladder, our rope ladder model makes explicit some of the challenges that occur within the process of participation. This recognizes the learning potential and collective growth that is inherent in challenges. While Arnstein’s ladder may have characterized slips such as moving down the ladder as veering into tokenism, our rope ladder envisions challenges, or knots, as further sources of tensile strength that can be freed up (when solved) and contribute to enhancing participation. In considering knotting and twisting in our HEALYAC, we implemented mechanisms to guide decision-making, such as quorum. For example, establishing a quorum for our YAC, and ensuring that these mechanistic processes are in place enables these young adults to stress their personal views on the topic at hand, whilst together as a YAC, it allows them to collectively make decisions and overcome these so-called knots. As a result of these meetings, these knots can be undone, thus increasing the strength and effectiveness of both our ladder and our YAC.

### 4.4. Looping

The inherent flexibility of the rope ladder means that it can be configured into positions that are beyond the scope of a traditional ladder. This provides an opportunity to connect youth participation within an adult partnership in ways that enable a ‘feedback loop,’ or a mechanism to establish accountability among youth and adult partners in an initiative. As shown in Figure 2d, the ladder can loop around the adult component of the partnership in ways that allow for iterative forms of engagement, communication, and support. The adult partners, represented by the tree, help to give structure to the rope ladder as it winds and loops around the trunk and branches. This looping allows for opportunities for the adult partners to frequently report back to the youth on how their input is being used, connecting the partnership in a feedback loop. This loop is essential to ensuring alignments between the goals of the youth participants and the adult partners. This looping and circling back illustrates the symbiosis of the adult–youth partnership and the ways they can work together to form, and reform, their relationship. 

In designing our YAC, we developed what we called a feedback loop, to ensure accountability between the HEAL and the YAC. This mechanism was built into our Terms of Reference and operationalized as a lab ‘report-back’ at every YAC meeting. The intention is that the HEAL is always accountable for how it addresses input from the YAC, and responsible for tracking and communicating about how, if, and when it implements YAC ideas. One of our youth YAC Development Team members reflects on the importance of the feedback loop in Box 3. We also embedded evaluation in our Terms of Reference in the form of dedicated evaluation points twice per year, so that members can inform and improve both the operations and the experience of the YAC, as well as to understand the impact of the YAC on HEAL research outcomes. The looping function is a mechanism to prevent tokenistic approaches to youth engagement and works, to ensure that youth participation can make a meaningful difference [1,3]. Building in evaluation measures from the beginning, so that there are metrics to assess not only the impact of youth participation, but to ensure that adult partners are accountable, is crucial to support an iterative process of adjustment while learning from successes and challenges [30].

Box 3 **Reflections on the feedback loop.**

Prior to this project, I had little to no knowledge regarding Youth Advisory Councils, so one of my first tasks was to review literature from other YACs, and similar organizations. During this period of the development process, my team and I became familiar with other Youth Advisory Councils within London, Ontario. One of the key principles which we discovered to be of utmost value for our Terms of Reference was that a bi-directional relationship must be present within the YAC. Essentially the members of the YAC and the HEAL benefit within this symbiotic relationship. This idea became the foundation of the Terms of Reference so that aspects of tokenism could be minimized within the HEALYAC.

One of our greatest applications of the symbiotic relationship came from the feedback loop. Which in essence was that after receiving the feedback from the Youth Advisory Council, the lab would be held responsible for keeping the YAC in the loop on how their feedback is being applied. This I think is the hallmark of our YAC model, as it emphasizes the values of equality and accountability in our model. As well as it ensures that the YAC members are genuinely involved and want to be further involved within the HEALYAC and with the operations of the HEAL.

~ Ahad Mahmood, Grade 12

## 5. Conclusions: Swinging into Action

By revealing our experiences in establishing a HEALYAC, we aimed to share the inspiring moments that we had together, as well as the pitfalls that we encountered, that led us to reflect on the participatory process in more detail. Evidence from our youth-driven process calls for a modified version of Arnstein’s ladder, one that is more adapted to youth’s needs and wants. As researchers, we have to consider that just because all young people are provided with the opportunity to be involved at any given stage of the research process, not all will want to participate at that highest level at all times. This fluid process of participation is constantly subject to change. The highest or last rung on the traditional ladder of participation is not necessarily an ideal or goal to be achieved, it is a temporary stage or moment in time throughout the research process, whereby youth move toward and away from this as they please. Therefore, we conclude that a new metaphor/model of participation is warranted, that addresses the complexity, flexibility, fluidity, and changing nature of participation throughout any participatory process. We propose as a potential answer the rope ladder model both for establishing and evaluating participatory processes with young people. 

In moving forward, we will evaluate the effectiveness of the HEALYAC over time, since our council will be retained over the long-term, and allow for young people to oversee and actively participate in the research process at various stages and times. This will help to fill the lack of research on the evaluation of the long-term effects of YACs and other types of youth participation, as well as to ensure that youth are engaged in evaluation processes—a gap that Hart has identified [35]. 

We conclude that the rope ladder concept is a more dynamic model for informing our YAC and has greater potential to establish a thriving relationship between youth and adults in a research context. This redistribution of power between the two groups fosters active youth participation and meaningful collaboration. The development of this particular YAC partnership offers promise for the generation of research that influences both health policy and practice. Our unified values as a co-investigative team allow us to better work towards the HEAL’s objective of developing healthy communities for young people. Importantly, the YAC provides valuable and meaningful opportunities for the youth involved, as our youth team members reflect, in conclusion, in Box 4.

Box 4 **Post-launch reflections on the YAC experience.**

Launching the YAC has been an incredible experience. It feels amazing to say that it will not only will it benefit our community, but will also benefit the members of the YAC by providing them with a unique research opportunity to build important experiences for their futures. If I could go back to where this development started and know what it would turn out to be, I would be astounded.

~ Suraj Paul

It really is amazing to see all our effort, finally being put into practice, as we are able to see our Youth Advisory Council in action. Thinking back to early January when we got this project, I never would’ve thought how I invested I would be in this project, and how fun the experience would be. I am eager to see how the YAC will continue to develop, and how its current and future members will shape it.

~ Ahad Mahmood

Running interviews and reading applications made me realize how many unique voices we have within our city, and I could not be more excited to see how they impact the research done at the lab. We hope that the council will run as smoothly as we planned it to be, but we are also expecting little bumps along the way, since mistakes are always a part of the learning process. I’m thrilled for other youth to receive an experience as incredible as the one I was lucky enough to obtain!

~ Krishna Arunkumar
